# Forecasting blood supply and demand under population aging: implications and challenges for healthcare resource allocation

**DOI:** 10.1186/s12913-026-14150-9

**Published:** 2026-02-12

**Authors:** Yueh-Mei Lin, Tsing-Fen Ho, Chao-Chun Ma, Ju-huei Chien

**Affiliations:** 1 Department of Medicine Laboratory, Jen-Ai Hospital, Taichung, 412224 Taiwan; 2 Department of Medical Laboratory Science and Biotechnology, Central Taiwan University of Science and Technology, 666 Buzih Road, Taichung, 40601 Taiwan; 3 Department of Research, Taichung Tzu-Chi Hospital, Buddhist Tzu-Chi Medical Foundation, 88 Fengxing Rd, Taichung, 427003 Taiwan

**Keywords:** Aging, Transfusion, Blood supply and demand, Forecasting, Demographic, Young first-time donor, SWOT analysis

## Abstract

**Background:**

Population aging poses major challenges to health-care systems by increasing the prevalence of chronic diseases and raising the demand for blood transfusion. Blood is essential in surgery, cancer therapy, and critical care, which are frequently used in older adults. However, declining birth rates and diminishing younger cohorts threaten the sustainability of the blood donor pool.

**Methods:**

In this study, we integrated demographic data from the Taiwanese household registration system, annual blood supply and demand data from the Taiwan Blood Services Foundation, and national population projection data from the National Development Council of Taiwan to forecast the supply-and-demand dynamics of blood in Taiwan through 2060.

**Results:**

An analysis of population pyramids revealed Taiwan’s progression toward a superaged society, with profound implications for blood supply management. It also revealed a marked reduction in the absolute number and proportion of young first-time donors. According to population projections, the number of individuals eligible to donate blood (aged 17–65 years) is expected to decrease from 16 million in 2025 to approximately 8.7 million by 2060. We identified a projected crossover point around 2027, when demand is expected to exceed supply. If current trends persist, this shortfall is forecasted to surpass 1 million units annually by 2060.

**Conclusions:**

The SWOT analysis further identified internal strengths and weaknesses, alongside external opportunities and threats. Based on this, some strategic directions were proposed to enhance recruitment, optimize utilization, adopt emerging technologies, and safeguard long-term sustainability. Ensuring blood availability is essential for healthy aging, as equitable access to transfusion supports medical interventions that preserve functional ability and improve quality of life in older adults. Overall, these findings highlight the urgent need for developing proactive donor recruitment strategies, reconsidering transfusion eligibility criteria, and enhancing patient management to ensure the availability of blood as a key resource.

**Supplementary Information:**

The online version contains supplementary material available at 10.1186/s12913-026-14150-9.

## Background

Blood transfusion is a key component of modern health care. It supports the management of trauma and hematologic and oncologic disorders and enables major surgery and palliative care. Ensuring a safe and sufficient blood supply requires a stable donor base, effective recruitment strategies, and efficient utilization practices. However, demographic transitions, particularly population aging and declining birth rates, pose growing challenges to the sustainability of blood supplies worldwide [1]. As populations age, the demand for blood products increases; for example, approximately 50% of individuals who require blood transfusion are those aged ≥ 60 years, indicating the disproportionate need for blood products in this demographic [2]. In addition to this unequal distribution, countries with rapidly aging populations have a shrinking pool of eligible donors alongside increasing transfusion requirements [3, 4]. This dual pressure reflects two concurrent trends: younger cohorts make fewer blood donations, whereas older populations, who are more likely to require blood transfusion because of a chronic illness or age-related procedures, expand in size [5]. High-income countries such as Japan, South Korea, and several European nations have already documented this phenomenon, reporting both reduced donor availability and increasing transfusion requirements [6–9].

Taiwan is currently undergoing one of the fastest demographic transitions worldwide [10]. According to the National Development Council of Taiwan, the country is expected to become a super-aged society by 2025, with more than 20% of its population aged ≥ 65 years [11]. This transition from an aged to a super-aged society within only seven years is substantially faster than that observed in Europe and the United States, where comparable transitions have typically spanned 20 to 50 years, and faster than those observed in other East Asian countries. Coupled with persistently low fertility rates, this rapid demographic shift is expected to fundamentally reshape the balance between blood supply and demand in Taiwan. This delicate balance is further influenced by developments in medical practice and healthcare utilization. In settings characterized by rapid population aging, regular monitoring of blood demand and the age distribution of both donors and recipients is essential for strategic planning to prevent shortages or unnecessary surpluses [6]. The COVID-19 pandemic has further highlighted the fragility of health systems and altered patterns of healthcare utilization and population health behaviors. Recent national-level analyses indicated that post-pandemic recovery is characterized by persistent structural shifts rather than a simple return to pre-pandemic baselines. In this context, essential health system resources—including national blood supply systems—may face compounded pressures arising from both demographic aging and evolving healthcare demand, underscoring the importance of long-term, policy-oriented forecasting [12]. Beyond demographic change alone, emerging evidence indicates that healthcare infrastructure and digital access substantially shape medical utilization among older adults. National-level analyses have shown that higher physician density and greater household internet access are associated with increased healthcare use in elderly populations [13]. In aging societies, such structural and technological factors may further amplify demand for medical interventions—including transfusion services—at the same time that the potential donor pool contracts due to declining working-age populations. In addition to demographic and structural determinants, the stability of blood supply systems also depends on human capital and organizational capacity. Recent evidence suggests that workforce competencies, organizational learning systems, and professional ethics are closely associated with adherence to quality and safety standards in healthcare organizations, underscoring the importance of “smart” organizational characteristics in maintaining system resilience. In this study, we utilized population survey data from the past decade, together with blood collection and utilization records from the Taiwan Blood Services Foundation, to forecast future changes in blood donation eligibility and demand through 2060.

## Methods

### Data sources

This study used three types of data sets: (1) demographic data from the Department of Household Registration, Ministry of the Interior, Executive Yuan, (2) annual blood supply and demand data published by the Taiwan Blood Services Foundation from 2010 to 2024; and (3) national population projection data from the National Development Council of Taiwan (2024 edition).

### Population pyramid construction

Raw data were aggregated into annual totals and organized into a time series format in Microsoft Excel (Microsoft, Redmond, WA, USA). All data were cleaned to ensure consistency in year-to-year reporting. Historical demographic data were compiled on the basis of records from the Department of Household Registration. Future population projection data were obtained from the National Development Council. These data sets were used to construct population pyramids illustrating age and sex distributions across different time points.

### Blood donor age distribution

In Taiwan, healthy individuals aged 17–65 years are eligible to donate blood. To facilitate blood donation and enhance donation services, the Taiwan Blood Services Foundation has progressively enhanced its blood management information system. This enhancement led to a steady improvement in the volume and quality of donor data available. In this study we analyzed the annual reports published by the Taiwan Blood Services Foundation between 2011 and 2023. Using these reports, we extracted and examined the age distribution of blood donors over the 13-year period from 2011 to 2023 to assess temporal changes in the proportion of donors across different age groups. We also drew future projections of blood supply by examining the medium population growth of national projection. These forecasts account for the rates of fertility, mortality, and net migration. In this study, the projected number of eligible donors was derived from population counts of individuals aged 17–65 years. Although this age-based criterion aligns with the current donor eligibility standards, it does not account for future changes in policy, health status, or donor recruitment strategies that may influence the actual donor pool.

### Forecasting method

To estimate future blood demand, we analyzed the demographic transitions projected in Taiwan over the period from 2025 to 2060. To validate the forecasting model, we retrospectively applied it to historical data obtained from annual reports published by the Taiwan Blood Services Foundation from 2011 to 2024. Actual blood supply records were also used to simulate predicted demand and evaluate model performance. An exponential smoothing model was applied to forecast both total blood demand and total blood donations. Forecasting was conducted using the “Forecast Sheet” feature in Microsoft Excel LTSC for Enterprise 2021, which implements a variant of exponential smoothing with additive error and trend components and no seasonality. This tool generates point forecasts together with 95% confidence intervals. To project future blood donations, we assumed that the average number of units donated per eligible donor would remain constant over time. Based on data from the preceding five years, the average donation intensity we estimated at 2.31 ± 0.05 units per donor. Under this assumption, a gradual decline in total blood donations from 2023 to 2060 was projected. This forecasting procedure involved selecting relevant time-series data (year versus number of blood units), specifying the forecast horizon (10–50 years), and exporting the predicted values and confidence intervals for subsequent analysis and visualization. Different baseline years were used across figures to reflect data availability and reporting formats. Specifically, demographic projections in Fig. 3A are presented at five-year intervals beginning in 2010, consistent with national population projection conventions, whereas Figs. 2 , 3B, and 4 are based on annual blood donation and utilization data available from 2011 onward.

**Fig. 1:**
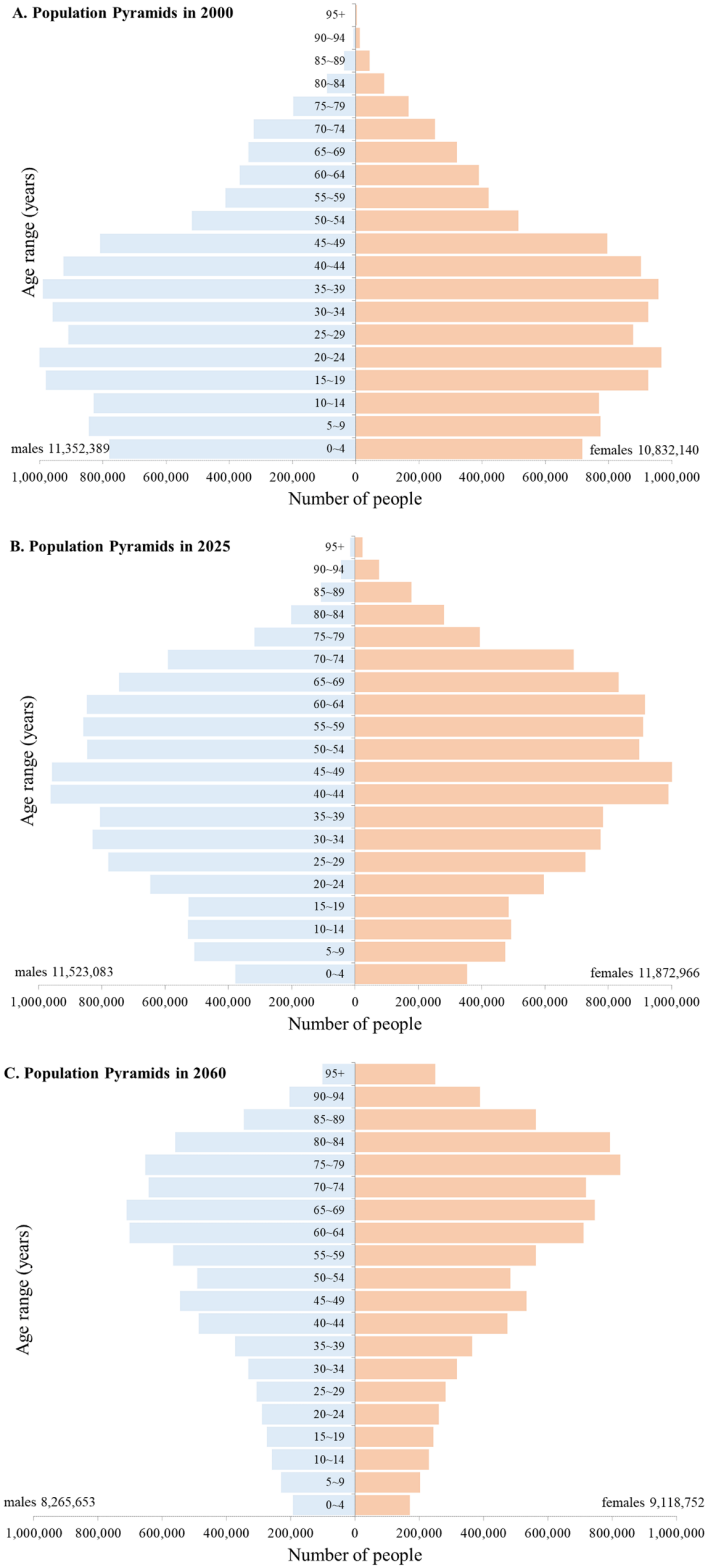
Population pyramids of Taiwan in 2000, 2025, and 2060, stratified by sex and age. This figure presents a comparative analysis of Taiwan’s population age structure across three time points: 2000 (historical), 2025, and 2060 (projected). The historical data for 2000 were obtained from the Department of Household Registration, Ministry of the Interior, Executive Yuan, and the population projections for 2025 and 2060 were derived from the 2024 forecasts of the National Development Council. Population is shown in 5-year age groups, with males displayed on the left (blue) and females on the right (Orange)

**Fig. 2:**
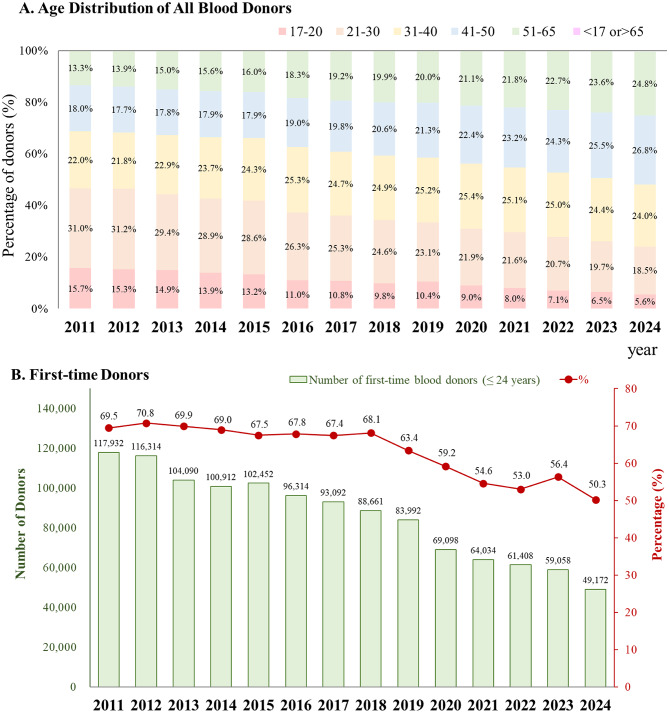
Donor population trends in Taiwan from 2011 to 2024. (**A**) Age distribution of all blood donors from 2011 to 2024. This stacked bar chart displays the annual percentage of blood donors across six age groups: 17–20 years, 21–30 years, 31–40 years, 41–50 years, 51–65 years, and < 17 or > 65 years. From 2011 to 2024, the proportion of younger donors (17–20 years) steadily declined, whereas the proportion of older donors, particularly those aged 51–65 years, gradually increased. (**B**) Proportion and number of first-time donors aged ≤ 24 from 2011 to 2024. Green bars represent the annual number of first-time blood donors aged ≤ 24 years (left *y*-axis), while the red line represents the percentage of these donors among all first-time donors each year (right *y*-axis). Both indicators demonstrate a sustained decline in youth engagement in first-time blood donation over the study period

**Fig. 3:**
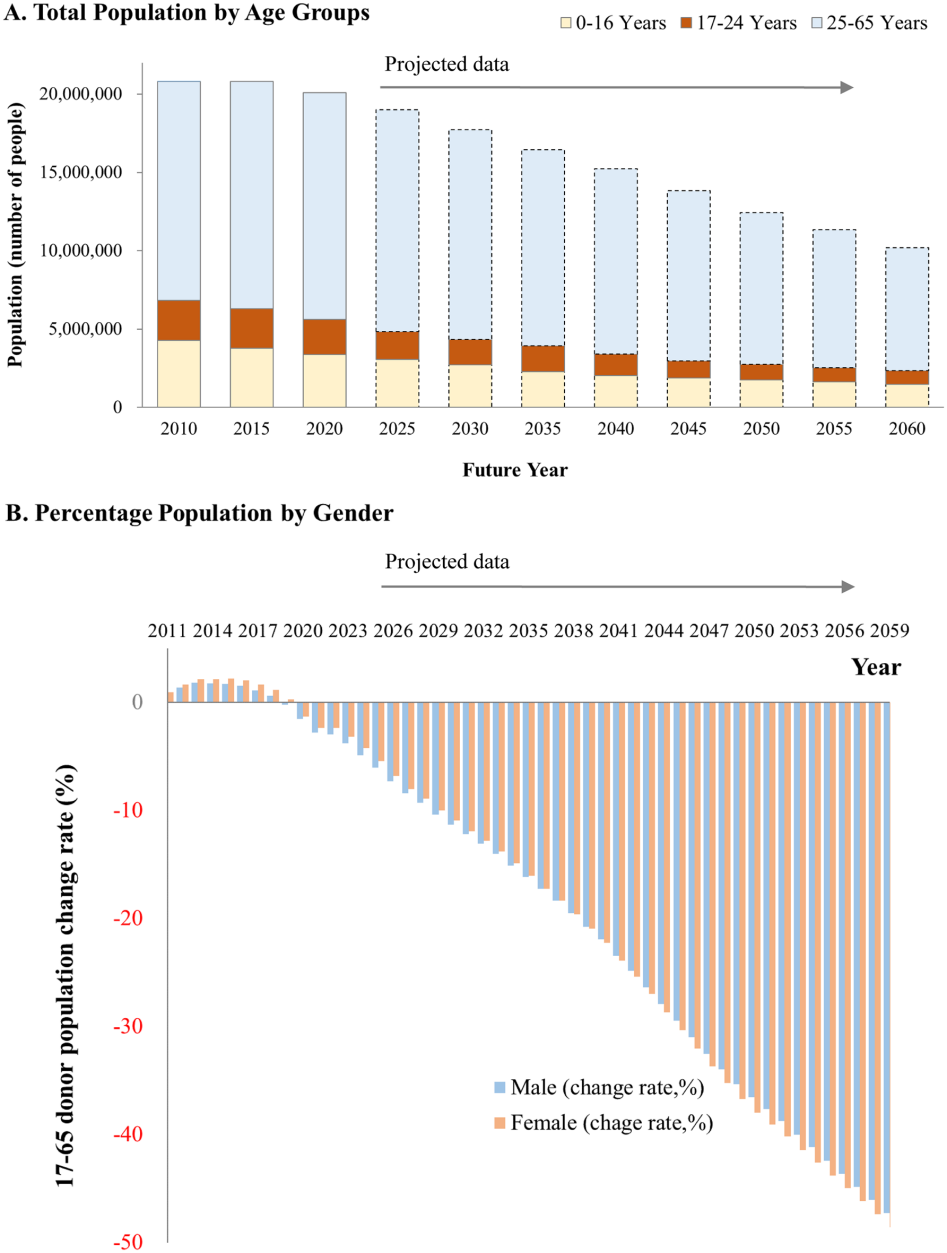
Projected changes in the population of eligible donors aged 17–65 years in Taiwan from 2010 to 2060. (**A**) Total population stratified by age (0–16 years, 17–24 years, and 25–65 years), illustrating trends in the non-eligible population, young donor pool, and core donor pool over time. Solid bars represent observed data, and dashed bars indicate projected data. (**B**) Projected percentage change in the eligible donor population (aged 17–65 years) stratified by sex. Bar represent the percentage change in the population of eligible males (blue) and females (Orange) donors relative to the 2010 baseline

**Fig. 4:**
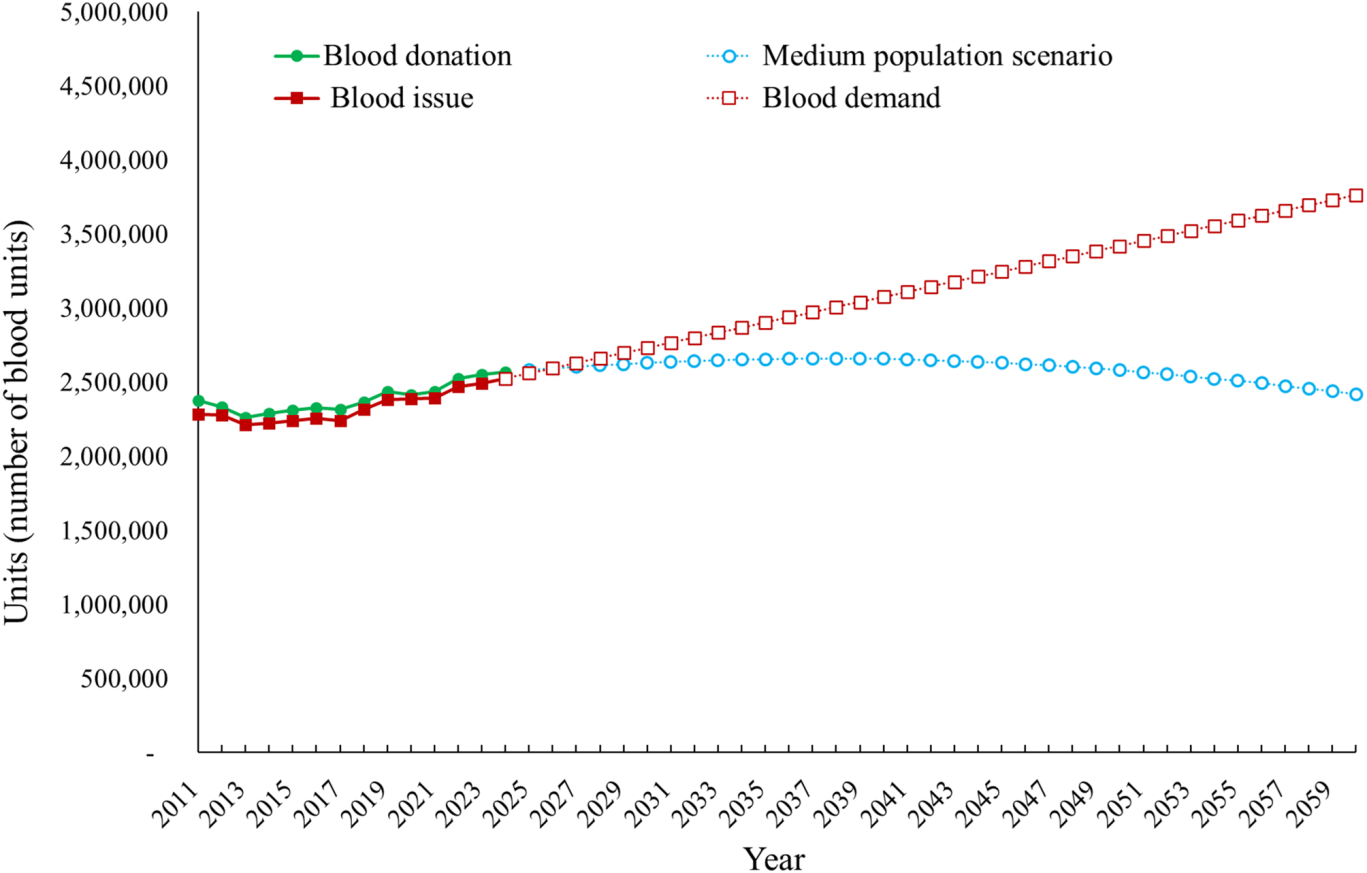
Historical and projected trends of blood supply and blood demand in Taiwan from 2011 to 2060. Solid lines represent historical data on blood donations and blood issue volumes from 2011 to 2024, and dashed lines represent projected estimates from 2025 to 2060. The green line with circles represents observed blood donation volumes, and the red line with squares represents observed blood issue volume. Blue open circles indicate projected blood supply under the medium population growth scenario, while red open squares indicate projected blood demand based on demographic projections and health-care utilization trends. All values are presented as the number of blood units donated or issued. The intersection of projected supply and demand curves indicates the anticipated onset of supply–demand imbalance

### Model validation

To evaluate the accuracy of the forecasting model, back-testing was conducted using historical data. Specifically, the exponential smoothing model was trained on data from 2011 to 2020 to generate forecasts for the period between 2021 and 2024. The forecasted values were then compared with the actual observed blood issue volumes reported by the Taiwan Blood Services Foundation. Model performance was assessed using standard error-based metrics, including the mean absolute error and root mean square error, together with visual inspection of observed versus fitted values to ensure model stability and plausibility. Overall, the forecasting model demonstrated acceptable predictive performance for the purpose of long-term trend projection.

## Results

### Population age structure by sex in 2000, 2025, and 2060

Figure 1 depicts a progressive demographic shift in the age distribution of the Taiwanese population from 2000 to 2060. In 2000, the population pyramid demonstrates a broad base and a narrow apex, which are characteristic of a young population with relatively high birth rates. By 2025, the base substantially narrows, indicating declining birth rates, whereas the upper segments of the pyramid expand, consistent with population aging. Projections for 2060 suggest an inverted pyramid shape, with a high concentration of older age groups and a sharp reduction in younger cohorts, indicating both advanced population aging and overall population decline. Across all time points, women consistently outnumber men in older age brackets, a pattern likely attributable to the longer life expectancy of women.

### Donor population trends

A major shift was observed in the age distribution of blood donors from 2011 to 2024. The proportion of donors aged 17–20 years steadily decreased from 15.7% in 2011 to 5.6% in 2024 (Fig. 2A). A similar downward trend was observed among donors age 21–30 years. By contrast, a uniform increase was observed in the proportion of donors aged 51–65 years, rising from 13.3% to 24.8% over the same period. During this period, the proportions of donors aged 31–40 and 41–50 years remained relatively stable, with only minor fluctuations observed. By 2023, individuals aged 41–65 years had accounted for more than half of the entire donor population, indicating a clear shift toward an aging donor base. During the same period, a uniform decline was observed in the absolute number and percentage of first-time blood donors aged ≤ 24 years (Fig. 2B). The number of young first-time donors decreased from 117,932 in 2011 to 49,172 in 2024, representing a reduction of 58.3%. Correspondingly, their proportion among all first-time donors decreased from 69.5% to 50.3%. Notably, this decline became faster after 2019, with a marked drop between 2019 and 2020 (from 83,992 to 69,098 donors). Throughout the study period, both the absolute number and proportion of young first-time donors markedly decreased. Overall, these findings underscore a significant and sustained reduction in the number of young first-time blood donors, highlighting a demographic shift that may necessitate targeted recruitment strategies to engage younger donors and ensure the long-term stability of blood supply.

### Long-term projections of the eligible blood donor population

Figure 3 presents the projected demographic shifts of the eligible blood donor population in Taiwan between 2010 and 2060. Panel A displays absolute population counts across three age groups: 0–16 years (future potential donors), 17–24 years (emerging donors), and 25–65 years (core donors). The overall population across all age groups is expected to decline over time, with the sharpest decline observed among donors aged 25–65 years. Although donors aged 17–65 years remain the primary source of blood donations, their proportion has started demonstrating a significant downward trajectory after 2020. The proportion of donors aged 17–24-years has also started demonstrating a steady decline, limiting the recruitment of new and young donors. Panel B depicts the rate of change in the proportion of eligible donors aged 17–65-years stratified by sex. In the early 2020s, the proportions of both male and female donors started exhibiting a steady and nearly identical decline. By 2040, these proportions are expected to decline by more than 20%, and by 2060, they are expected to decline by more than 50% relative to the 2010 baseline. Overall, these findings highlight a key demographic challenge for the blood services system of Taiwan: the dual impact of an aging population and shrinking donor pool across both male and female donors. To address this challenge, strategic interventions are urgently required to maintain the donor population.

### Estimated future blood demand and blood donations

Analysis of blood demand and supply trends from 2011 to 2060 revealed a projected decline in blood donations in a medium population growth scenario. Between 2011 and 2024, both blood donations and blood issue volumes in Taiwan remained relatively stable, with annual units ranging from approximately 2.3 to 2.5 million. A slight upward trend was observed starting around 2020, with blood donations and blood issue volumes converging at approximately 2.5 million units by 2024. Projections based on a medium population growth scenario indicate that blood donations will peak around 2027 and then gradually decline, falling below 2.5 million units by 2060. By contrast, projected blood demand is expected to steadily increase, surpassing 3 million units by 2037 and reaching more than 3.6 million units by 2060. This change represents a 40% increase relative to 2024, with an average annual growth rate of approximately 1.2%. Notably, the gap between blood supply and demand is expected to significantly increase after 2027, indicating a potential shortage in supply if these current trends continue unabated.

## Discussion

This study provides integrated projections of blood supply and demand in Taiwan as the country rapidly transitions into a super-aged society. Several key insights emerge from our findings. First, Taiwan’s total population is projected to decline substantially while population aging accelerates, reshaping both the size and age structure of the potential donor pool (Fig. 1). Second, the donor population itself is aging, as reflected by the marked and sustained decline in the proportion of young donors over the past decade (Fig. 2). Third, the number of eligible blood donors is projected to decrease significantly in the coming decades, posing a structural challenge to the long-term stability of the blood supply (Fig. 3). Finally, our model identifies a critical crossover point around 2027, when projected blood donations are no longer sufficient to meet transfusion demand (Fig. 4). Collectively, these trends underscore the urgency of strategic, system-level planning to ensure the sustainability of blood services in Taiwan (Table 1).

**Table 1:** SWOT analysis and corresponding strategic responses for Taiwan’s blood supply system

		Strengths	Internal factors	Weaknesses	Internal factors
		• Established blood supply and safety system • Strong culture of voluntary blood donation • Advanced medical technology and transfusion safety		• Blood product wastage (e.g., over-ordering, expiration) • Low engagement of younger donors • Lack of real-time, cross-hospital inventory systems	
**Opportunities**	External factors	**S/O Strategies**		**W/O Strategies**	
• AI and big data for supply-demand forecasting • International collaboration and best practices • Emerging technologies (synthetic blood, cell therapies) • Integration with long-term care and public health policies		• Apply AI-driven demand forecasting within Taiwan’s blood system • Expand voluntary donation through digital platforms, peer networks, campuses, and health education • Leverage international collaboration for synthetic blood and advanced medical technologies • Promote intraoperative autotransfusion and minimally invasive surgical techniques		• Enhance education and outreach to increase youth participation in blood donation • Build AI-supported, real-time cross-hospital inventory systems • Adopt international best practices to reduce operational inefficiencies • Develop proactive donor recruitment strategies, including healthy older adults • Reevaluate transfusion eligibility criteria	
**Threats**	External factors	**S/T Strategies**		**W/T Strategies**	
• Declining eligible donors (17–65 years) • Rising demand from population aging • Vulnerability to pandemics/disasters • Changing social values affecting donation		• Utilize strong infrastructure to maintain blood supply during emergencies • Promote donor retention to counteract a shrinking donor pool • Improve preservation technologies to extend shelf life and alleviate shortages • Implement patient blood management, optimize surgical techniques, and enforce stricter transfusion guidelines		• Reduce blood wastage through optimized transfusion practices • Increase donor engagement to offset population decline • Strengthen contingency planning for pandemics and disasters	

SWOT: strengths, weaknesses, opportunities, and threats; AI, artificial intelligence

Blood transfusion remains a cornerstone of modern medical care, particularly for older adults undergoing joint replacement surgery [14, 15], cancer treatment, and palliative care [16]. As societies age, transfusion support becomes increasingly central to maintaining functional independence and quality of life in later years. Failure to secure an adequate blood supply risks compromising access to essential medical services for older populations and undermining the broader goals of healthy aging. From a global perspective, population aging is not unique to Taiwan. France was the first country to enter an aging society in the mid-19th century, while Japan became the world’s first superaged society in 2005 [17]. Taiwan is projected to reach superaged status by 2025 [18], but the pace of this transition is among the fastest globally. Notably, Taiwan will complete the shift from an aging to a super-aged society within a markedly compressed timeframe, leaving limited opportunity for health systems to adapt incrementally. Specifically, Taiwan will have taken only 25 years to transition from an aging society to an aged society and only 7 years to transform from an aged society to a superaged society [19]. Our findings align with patterns reported in other high-income societies undergoing rapid demographic aging. Across Japan, South Korea [17], and multiple European countries, persistently low fertility combined with increasing life expectancy has progressively narrowed the pool of eligible blood donors while simultaneously expanding transfusion demand among older recipients. In addition, evidence from the Netherlands and other settings indicates that donor participation is strongly influenced by structural and organizational factors—such as accessibility and system design—rather than altruistic motivation alone, suggesting that institutional design plays a critical role in sustaining donor pools in aging societies [20]. Recent international comparisons further indicate that several high-income countries permit blood donation beyond 65 years of age under specific health conditions (Appendix Table A1), highlighting the potential flexibility of donor eligibility policies in response to population aging. Recent literature also emphasizes the potential strategic role of retaining and re-engaging older donors as a stabilizing force in aging societies, challenging traditional youth-centered recruitment paradigms and underscoring the need for adaptive donor policies [21].

Previous research has indicated a strong link between future blood supply and demographic characteristics [22]. In this study, we analyzed demographic data obtained from the Taiwanese household registration system for the period between 2011 and 2024, and we examined donor records maintained by the Taiwan Blood Services Foundation. We observed that the blood donation patterns in Taiwan are associated with population structure, consistent with the aforementioned finding. As depicted in Fig. 2A, the proportion of donors aged 17–20 years steadily decreased from 15.7% in 2011 to 5.6% in 2024, and the proportion of donors aged 17–30 years also uniformly decreased, accompanied by an increase in the proportion of middle-aged donors (51–65 years). Although this growing contribution of middle-aged donors provides short-term stability [21], it raises long-term concerns, particularly because this cohort is at a high deferral risk of age-related conditions or medication use and is eventually expected to become ineligible to donate [22, 23]. Simultaneously, the number of first-time donors has markedly decreased. Specifically, the number of first-time donors aged ≤ 24 years substantially decreased from 117,932 (69.5%) in 2011 to 49,172 (50.3%) in 2024, reflecting a reduction of 58.3% (Fig. 2B). First-time blood donors are key contributors to the blood donor base [24]. Traditionally, blood donors recruitment efforts have targeted younger populations because they are assumed to be healthier and able to donate blood for longer periods [25, 26]. Furthermore, the decline observed in the number of first-time donors reflects multiple aspects, including academic and economic pressures, reduced school-based drive, and the impact of COVID-19, which disrupted recruitment channels and imposed additional deferrals [27, 28]. Together, these trends exacerbate existing challenges by combining declining participation of young donors, who represent the future of the donor base, and the increasing reliance on older donors, whose capacity may become limited. In recent years, the proportion of young blood donors in Taiwan has markedly declined, whereas reliance on middle-aged donors has increased. Although older donors often demonstrate higher return rates and more uniform donation patterns, they are also at a greater risk of becoming ineligible due to the possibility of age-related issues [29]. In addition, an aging recipient population is projected to drive a sustained increase in blood demand [21]. This evolving landscape of blood supply and demand necessitates the development of progressive strategies to ensure blood transfusion sufficiency and safety in the upcoming decades.

Figure 3 illustrates major demographic transitions that are expected to shape Taiwan’s future blood donor base. After remaining stable or even increasing before 2020, the population of both younger donor (17–24 years) and core donors (25–65 years) are projected to steadily decline after 2020, with an accelerating downward trend over time. By 2059, the population of both male and female eligible donors are projected to have decreased by approximately 50%, presenting difficulties in maintaining a stable blood supply. Similar patterns have been observed in other high-income countries, where population aging has resulted in a decrease in the number of core donors and limited the inflow of first-time younger donors [30, 31]. The parallel decline in the number of both male and female donors suggests that this trend is not sex-specific but rather reflects broader structural demographic transitions. Similar non-sex-specific downward trends have been reported in population-based donor projections in Europe and Japan [8, 32]. These findings underscore the need for population-wide interventions and continuous monitoring of donor demographics.

Integrating donor population forecasts with blood utilization projections is essential for anticipating future challenges and shaping effective policy responses. The steady increase observed in blood demand reflects the anticipated rise in age-related medical procedures, chronic disease management requirements, and surgical interventions [5]. As shown in Fig. 4, blood demand is projected to continue increasing, driven by population aging and associated medical needs, thereby heightening the risk of a sustained imbalance between supply and demand. Although a slight recovery has been observed in both blood donations and blood issue volumes during the period from 2020 to 2023—likely reflecting post-pandemic normalization and targeted recruitment efforts—blood supply is projected to begin declining again around 2030. This projected inflection point corresponds to major demographic transitions, including Taiwan’s rapid population aging and persistently low birth rates, which have long-term implications for health-care resource planning. Extending the projection horizon to 2060 allows these structural demographic effects to be fully captured and provides a policy-relevant perspective beyond short-term operational forecasting. Importantly, projections extending to 2060 should be interpreted as a strategic, long-horizon boundary scenario that illustrates the cumulative impact of demographic aging on the blood supply–demand balance, rather than as a deterministic forecast. While long-term projections are inherently subject to greater uncertainty, the direction and timing of the projected divergence between blood supply and demand are largely driven by robust demographic processes that remain consistent across plausible population scenarios. By 2060, the annual imbalance between demand and supply is projected to exceed 1 million units if donation levels are not maintained or improved. From a policy standpoint, the next 5 years nonetheless represent a crucial window for intervention, as early mitigation strategies implemented during this period may substantially influence longer-term trajectories. Together, these findings highlight the complementary roles of short-term monitoring for operational planning and long-horizon projections for identifying structural risks and informing strategic blood supply policies. In this regard, regular model updating and shorter-term reassessments over policy-relevant horizons (e.g., 10–20 years, such as 2035–2045) are essential to reflect evolving donor behaviors, clinical practices, and health system conditions, and to support actionable decision-making. In addition, over the next 10–20 years, policy and societal changes could alter long-term donor availability and the future blood supply landscape. On the demand side, strategies that promote healthy aging, strengthen chronic disease management and community-based care, and expand patient blood management programs may help reduce avoidable transfusion needs and mitigate future demand growth. Together, these considerations reinforce the importance of regular model updating and shorter-term reassessments (e.g., 2035–2045) to support policy implementation and operational decision-making under evolving demographic and clinical conditions. Several simplifying assumptions underlie the present projections. Donor eligibility age limits and the average number of donated units per donor were assumed to remain constant over time to focus on the demographic drivers of supply–demand imbalance. In practice, policy adjustments—such as extending eligibility to healthy older adults or increasing donation frequency through targeted retention strategies—could partially offset projected supply declines. Conversely, stricter eligibility criteria or reduced donation intensity would likely exacerbate future shortages. These projections should therefore be interpreted as policy-relevant baseline scenarios rather than precise forecasts. In addition, patient blood management and clinical transfusion practices could not be incorporated directly into the forecasting model because consistent national-level indicators and time-series data were not available; therefore, they are more appropriately framed as follow-up strategies and decision-support levers that may mitigate future demand growth and should be monitored alongside updated projections.

To address the aforementioned challenges, multifaceted strategies are required to both replenish the donor pool and ensure the sustainability of the blood supply. The SWOT framework, summarized in Table 1, guided the development of four categories of strategies by linking internal strengths and weaknesses with external opportunities and threats [33]. S/O strategies build on Taiwan’s strong infrastructure, culture of voluntary donation, and advanced medical technologies by applying AI-driven forecasting, expanding recruitment through digital platforms and health education, strengthening international collaboration for synthetic blood, and promoting intraoperative autotransfusion or minimally invasive surgery. W/O strategies address internal gaps by enhancing outreach to younger donors, developing a real-time cross-hospital inventory system, adopting international best practices, and re-evaluating donor eligibility to safely include healthy older individuals. Meanwhile, S/T strategies leverage existing strengths to counter demographic and systemic threats by maintaining supply during emergencies, improving preservation methods [34], and implementing patient blood management with stricter transfusion guidelines [35]. Finally, W/T strategies mitigate vulnerabilities by reducing wastage [36], improving donor engagement, strengthening contingency planning for disasters [37], and reassessing transfusion eligibility criteria [38]. From a translational and policy implementation perspective, these strategies can be further organized into three priority packages to support decision-making. The first package focuses on data integration and demand forecasting, including AI-assisted prediction and real-time inventory management, representing a short- to medium-term priority primarily led by national blood services and health authorities. The second package emphasizes donor recruitment and retention, particularly among younger populations and healthy older adults, requiring coordinated medium-term efforts involving blood centers, educational institutions, and community organizations. The third package addresses clinical demand management and system resilience, encompassing patient blood management, optimized transfusion practices, preservation technologies, and disaster preparedness, which constitute longer-term priorities jointly implemented by hospitals, clinicians, and policymakers. Collectively, these integrated and staged approaches highlight how technological innovation, institutional coordination, and clinical optimization can be aligned to enhance the resilience of Taiwan’s blood supply system. By combining proactive donor policies with evidence-based demand management, Taiwan can better adapt to demographic shifts and secure a sustainable blood supply for future healthcare needs.

### Limitations and future directions

This study has several limitations. First, our projections are based on national demographic estimates and historical transfusion data, which may not fully capture unforeseen changes in health-care practices, donor eligibility criteria, or advancements in medical technology. Second, the forecasting approach was constrained by the limited length and annual resolution of available historical blood donation and issuance data. While advanced time-series and machine-learning models—such as ARIMA-based or hybrid deep-learning frameworks—may offer improved short-term predictive performance in other contexts, their application to multi-decade projections with sparse data can lead to unstable or implausible extrapolations. Accordingly, this study prioritized model transparency, interpretability, and long-term stability over short-term predictive optimization to support macro-level planning. Third, the model does not explicitly incorporate potentially influential clinical and policy determinants, such as patient blood management implementation intensity or changes in transfusion thresholds, because consistent national-level time-series indicators were not available. Finally, although Taiwan represents a valuable case study because of its rapid population aging dynamics and universal health-care coverage, direct generalization to other health-care systems should be made with caution. Future studies should incorporate higher-frequency and longer time-series data, evaluate dynamic policy interventions such as patient blood management programs, and conduct periodic recalibration and shorter-horizon reassessments to support policy implementation as demographic conditions and clinical practices evolve. 

## Supplementary Information

Below is the link to the electronic supplementary material.

Supplementary Material 1

## Data Availability

All data analyzed in this study are either publicly available from government websites (Ministry of the Interior and National Development Council of Taiwan) or are available upon reasonable request with permission from the Taiwan Blood Services Foundation.
