# Supplementary material for: Forecasting blood supply and demand under population aging: implications and challenges for healthcare resource allocation
Source: BMC Health Serv Res. 2026 Feb 12;26:369. doi: 10.1186/s12913-026-14150-9 (PMC12997664; doi:10.1186/s12913-026-14150-9)
Supplement: Supplementary file 1 — Supplementary Material 1 [file 12913_2026_14150_MOESM1_ESM.docx]

**Appendix Table A1**. Blood donor age eligibility limits in selected countries

| **Country/Region** | **Minimum age** | **Upper age limit (first-time / repeat donors)** | **Population aged ≥65 years (%)** | **Reference / official source** |
| --- | --- | --- | --- | --- |
| Taiwan | 16 (parental consent required if under 17) | Up to 65; ages 66–70 eligible with medical assessment | 19.6% (2025) | Taiwan Blood Services Foundation  https://www.blood.org.tw/files/file_pool/1/0P076362309725494200/b572e88e-dd66-4b15-9ad5-97c9e0ed8f97.pdf |
| Japan | 17–18 (depending on donation type) | Common upper limit around 69; varies by component and guidelines | 29.8% (2024) | Japanese Red Cross Society  https://www.jrc.or.jp/english/pdf/BloodServices2025.pdf |
| South Korea | 18 | 65+ eligible only with prior donation history and health criteria | 20.3% (2025) | Korean Red Cross (KRC)  https://smart.bloodinfo.net:59979/emi4.do?lang=en |
| China (Mainland) | 18 | National law suggests 18–55; local rules may extend limits | 12.6% (2020) | Red Cross Society of China  https://www.nhc.gov.cn/fzs/c100048/201808/ea9fb73f399d4cedb1b3438825646505.shtml |
| Hong Kong | 16 (parental consent required) | First‑time up to 65; repeat donors 66–75 with medical clearance | 22.7% (2024) | Hong Kong Red Cross  https://www5.ha.org.hk/RCBTS/eligibility |
| Thailand | 17 (parental consent if <18) | Typical range 17–70; first‑time donors often <60 | 15.4% (2024) | Thai Red Cross Society  https://thaibloodcentre.redcross.or.th/donor-eligibility/ |
| Singapore | 16 (parental consent required) | First‑time up to 60; possible extension for regular donors | 20.7% (2024) | Singapore Red Cross  https://redcross.sg/give-blood/can-i-donate-blood.html#:~:text=To%20donate%20blood%20in%20Singapore%2C%20you%20must,menstrual%20flow%20*%20Have%20recently%20traveled%20overseas |
| India | 18 | Standard 18–65 | 6.7% (2020 Census data) | Indian Red Cross Society  https://www.indianredcross.org/ircs/program/bloodbank/camps/#whocangiveblood |
| Australia | 18 | First‑time up to 75; regular donors may continue beyond 75 with assessment | 17.7% (2024) | Australian Red Cross  https://www.lifeblood.com.au/faq/eligibility?query=DONATION+AGE |
| New Zealand | 16 | New donors 16–71; repeat donors up to ~81 with approval | 17.2% (2024) | New Zealand Blood Service (NZBS)  https://www.nzblood.co.nz/become-a-donor/am-i-eligible |
| United States | 16–17 (state‑  dependent) | No upper age limit if healthy | 17.4% (2023) | The American National Red Cross  https://www.redcrossblood.org/donate-blood/how-to-donate/eligibility-requirements.html |
| Canada | 17 | No upper age limit for repeat donors; some limits for first‑time donors | 19.8% (2024) | Canadian Blood Services  https://www.blood.ca/en/blood/am-i-eligible-donate-blood/eligibility |
| United Kingdom | 17 | First‑time up to 65; repeat donors up to 70–75 | 19.5% (2024) | NHS Blood and Transplant (NHSBT)  https://www.blood.co.uk/who-can-give-blood/ |
| Germany | 18 | Typical upper limit 68–70; >60 requires medical evaluation | 23.2% (2024) | German Red Cross (DRK)  https://www.uniklinik-freiburg.de/itg-en/blood-donation.html |
| France | 18 | Up to ~70 for whole blood; stricter for first‑time apheresis donors | 22.1% (2024) | Etablissement Français du Sang (EFS)/ service-public  https://www.service-public.gouv.fr/particuliers/actualites/A18431?lang=en |
| Italy | 18 | 18–65; extension to 70 with physician approval | 24.6% (2024) | AVIS (Associazione Volontari Italiani Sangue)  Centro Nazionale Sangue (National Blood Centre) provides guidelines.  https://www.centronazionalesangue.it/en/how-to-become-a-donor/ |
| Spain | 18 | First‑time up to ~60; repeat donors 65–70 with clearance | 21.2% (2024) | Red Cross (Cruz Roja) and the Blood Transfusion Service (BTS) in your region (e.g., Banc de Sang i Teixits for Catalonia, Centro de Transfusión de Valencia for Valencia).  https://www.bancsang.net/en/donations/blood/ |
| Netherlands | 18 | Standard 18–65; extended donation possible for repeat donors | 20.2% (2023) | Sanquin is the official blood bank in the Netherlands, handling collection and distribution, not the Red Cross, though the Red Cross (Rode Kruis) is involved in donor recruitment in Belgium.  https://www.sanquin.nl/en/donors/about-donating/can-i-become-a-blood-donor |
| Sweden | 18 | Most apply 18–60; older repeat donors accepted under evaluation | 20.6% (Dec 2024) | Stockholm/Uppsala via geblod.nu and regional blodcentralen  https://geblod.nu/om-blodgivning/english/ |
| Switzerland | 18 | First‑time up to 60; repeat donors up to ~75 depending on canton. | 20.0% (2024) | Swiss Red Cross (SRC)  https://en.blutspende.ch/en/information-donors/faq-blood-donation |

Population aged ≥65 years refers to the proportion of the total population aged 65 years and older in the indicated year, based on official national statistics.
